# Longitudinal Assessment of Amyloid Pathology in Transgenic ArcAβ Mice Using Multi-Parametric Magnetic Resonance Imaging

**DOI:** 10.1371/journal.pone.0066097

**Published:** 2013-06-19

**Authors:** Jan Klohs, Igna Wojtyna Politano, Andreas Deistung, Joanes Grandjean, Anna Drewek, Marco Dominietto, Ruth Keist, Ferdinand Schweser, Jürgen R. Reichenbach, Roger M. Nitsch, Irene Knuesel, Markus Rudin

**Affiliations:** 1 Institute for Biomedical Engineering, ETH and University of Zurich, Zurich, Switzerland; 2 Neuroscience Center Zurich, University of Zurich and ETH Zurich, Zurich, Switzerland; 3 Medical Physics Group, Institute of Diagnostic and Interventional Radiology I, Jena University Hospital – Friedrich Schiller University Jena, Jena, Germany; 4 Seminar für Statistik, ETH Zurich, Zurich, Switzerland; 5 Division of Psychiatry Research, University of Zurich, Zurich, Switzerland; 6 Institute of Pharmacology and Toxicology, University of Zurich, Zurich, Switzerland; Oregon Health & Science University, United States of America

## Abstract

Magnetic resonance imaging (MRI) can be used to monitor pathological changes in Alzheimer's disease (AD). The objective of this longitudinal study was to assess the effects of progressive amyloid-related pathology on multiple MRI parameters in transgenic arcAβ mice, a mouse model of cerebral amyloidosis. Diffusion-weighted imaging (DWI), T_1_-mapping and quantitative susceptibility mapping (QSM), a novel MRI based technique, were applied to monitor structural alterations and changes in tissue composition imposed by the pathology over time. Vascular function and integrity was studied by assessing blood-brain barrier integrity with dynamic contrast-enhanced MRI and cerebral microbleed (CMB) load with susceptibility weighted imaging and QSM. A linear mixed effects model was built for each MRI parameter to incorporate effects within and between groups (i.e. genotype) and to account for changes unrelated to the disease pathology. Linear mixed effects modelling revealed a strong association of all investigated MRI parameters with age. DWI and QSM in addition revealed differences between arcAβ and wt mice over time. CMBs became apparent in arcAβ mice with 9 month of age; and the CMB load reflected disease stage. This study demonstrates the benefits of linear mixed effects modelling of longitudinal imaging data. Moreover, the diagnostic utility of QSM and assessment of CMB load should be exploited further in studies of AD.

## Introduction

Alzheimer's disease (AD) has a complex pathophysiology with pathomorphological hallmarks of the disease being the misfolding of amyloid-β (Aβ) protein and hyperphosphorylation of the tau protein. These pathological changes are associated with the aggregation of Aβ plaques, neurofibrillary tangles, neuronal degeneration and neuroinflammation [1]. In addition, cerebrovascular dysfunction has been implicated in the etiology of the disease [2], [3]. Transgenic mouse models have been engineered to investigate the pathophysiology of AD [4], [5], where several mouse lines express the mutant genes for human amyloid precursor protein (APP) and presenilin, responsible for the proteolytic processing of APP. These transgenic mice develop Aβ aggregates (senile plaques) in the brain parenchyma in an age-dependent manner. Some strains show a variable degree of Aβ deposition at the cerebral vasculature and vasculopathy [6]. Despite the fact that transgenic mouse models do not completely replicate AD, they have become indispensible for studying disease mechanism and drug discovery.

Over the past years, a number of studies have emerged that use magnetic resonance imaging (MRI) for the phenotyping of transgenic mouse models of AD [7], [8]. Anatomical scans have demonstrated increased rates of brain atrophy in transgenic mice compared to wild type (wt) mice [9], [10]; and pathological alterations in brain connectivity [11]. Diffusion-weighted imaging (DWI) [12]–[14] and MR relaxometry [15]–[18] have revealed regional changes in tissue microstructure and composition. In addition, cerebrovascular dysfunction is characterized by cortical hypoperfusion [19], [20], reduced vascular reactivity [21], and the occurrence of cerebral microbleeds (CMBs) [22]. While MRI enables repetitive assessment of an animal and thus allows following an MRI parameter over the course of the disease, most studies were conducted as cross-sectional studies involving two or more age groups. There are only very few longitudinal studies [9], [17], although such a study design can increase statistical power by reducing the confounding effects of between-subject variability compared to cross-sectional studies [23]. Moreover, longitudinal studies can establish the dynamics of a parameter over the disease course, an important feature to evaluate the prognostic value of a parameter. Common problems in longitudinal studies are missing data due to drop-outs and non-uniform timing [17], [24], which significantly hampers the ability to draw the trajectories of parameters for individual animals. Hence, standard statistical analysis of longitudinal data like repeated measures ANOVA and cross-sectional (General Linear Model based) analysis of summary measurements cannot be used as they require balanced data sets [24]. Recently, linear mixed effects (LME) modelling has been proposed for the analysis of longitudinal studies where data sets can be unbalanced with regard to the measurement time [24], [25]. Moreover, this statistical approach can account for (several) effects unrelated to the disease pathology which cannot be performed with conventional statistical tests.

In the current study, we longitudinally assessed the effects of progressive amyloid-related pathology on multiple MRI parameters in transgenic arcAβ mice, a mouse model of cerebral amyloidosis [26], and used LME modelling for MRI data analysis. The arcAβ mouse develops age-dependent Aβ accumulation in the brain parenchyma as well as at cerebral vessels [6], [20], [22], [26], which makes it an ideal model to follow changes in structural integrity and vascular function induced by the Aβ pathology with MRI. Changes in tissue composition and structural alterations were monitored with DWI and T_1_-mapping. In addition to these established techniques, we applied quantitative susceptibility mapping (QSM), a novel technique that analyzes phase shifts caused in the MRI signal by local changes in magnetic susceptibility [27]–[29]. To assess impairment of blood-brain barrier (BBB) integrity, we applied dynamic contrast-enhanced MRI (DCE-MRI). CMB load, as a sign of severe vascular dysfunction, was assessed with susceptibility weighted imaging (SWI) [30], [31] and QSM, as paramagnetic heme-iron leads to pronounced local changes in the magnetic susceptibility. In this study, we found that factors unrelated to the disease pathology have a strong effect on all MRI parameters and that LME modelling can account for these factors.

## Materials and Methods

### Animals and study design

All procedures conformed to the national guidelines of the Swiss Federal act on animal protection and were approved by the Cantonal Veterinary Office Zurich (Permit Number: 172–2008). Transgenic arcAβ mice and wt littermates of either gender (Division of Psychiatry Research, University of Zurich) were used. Animals were kept at standard housing conditions (temperature 20–24°C, relative humidity minimum 40%, light/dark cycle 12 h) with water and food being provided ad libitum. Animals were assessed serially from 5 to 21 months of age, where a first batch of animals entered the study at 5 months of age and a second batch at 15 months of age (see Table 1). Each scan session consisted of two sets of measurements using MRI systems of different field strength: anatomical reference data, DWI, T_1_-mapping and DCE-MRI data were acquired at 4.7 T, whereas anatomical reference and gradient recalled echo (GRE) data were collected at 9.4 T for computing GRE magnitude images, SWI and QSM; taking advantage of the increased signal-to-noise ratio at the higher magnetic field strength. Anatomical reference images were used for planning of subsequent image acquisitions. Between the two assessments animals had one week of recovery. When animals needed to be euthanized after scanning, brains were collected for Aβ immunohistochemistry. The number of brains that were investigated with histology were n = 2 (scan1), n = 1 (scan2), n = 4 (scan 3), and n = 1 (scan5), respectively.

**Table 1:** 

	scan1	scan2	scan3	scan3	scan4	scan4	scan5	scan5
	batch1	batch1	batch1	batch2	batch1	batch2	batch1	batch2
**group size**								
wt	11	10	10	8	8	5	6	4
arcAβ	13	11	10	7	5	3	4	2
**age** *								
wt	4.8±0.4	9.4±0.4	13.3±0.4	15.0±1.1	17.7±0.4	18.4±0.1	21.1±0.5	22.0±0
arcAβ	4.8±0.4	9.7±0.4	13.4±0.2	14.9±1.2	17.8±0.01	18.4±0.03	21.2±0.2	21.9±0.04

*at entering first assessment of each scan session; values are given as mean±SD.

### MRI hardware and animal preparation

The first set of measurements were acquired on a Bruker PharmaScan 47/16 (Bruker BioSpin GmbH, Ettlingen, Germany) operating at 4.7 T and equipped with a volume resonator operating in quadrature mode for excitation and a four element phased array surface coil for signal reception. Anesthesia was induced using 3% isoflurane (Abbott, Cham, Switzerland) in a 4∶1 air/oxygen mixture. During MRI mice were spontaneously breathing under isoflurane anesthesia (1.5%).

The second set of measurements were acquired on a Bruker BioSpec 94/30 (Bruker BioSpin GmbH) small animal MR system operating at 9.4 T. The system was equipped with a cryogenic quadrature RF surface probe (Bruker BioSpin AG, Fällanden, Switzerland). Mice were endotracheally intubated and mechanically ventilated during measurements with 90 breaths/minute while applying a respiration cycle of 25% inhalation and 75% exhalation (MRI-1 Volume Ventilator, CWI Inc., Ardmore, USA) using 1.2% isoflurane. Fieldmap-based shimming was performed using the automated MAPshim routine to reduce field inhomogeneities.

During both scan sessions body temperature was monitored with a rectal temperature probe (MLT415, ADInstruments, Spechbach, Germany) and kept at 36.0±0.5°C using a warm-water circuit integrated into the animal support (Bruker BioSpin GmbH).

### Sequence parameters for the first set of measurements performed at 9.4T

1. Anatomical reference data were acquired by applying a spin-echo (SE) sequence (Rapid Acquisition with Relaxation Enhancement [RARE]) with TE/TR = 38.6/4200 ms, RARE factor  = 8, in axial, sagittal and horizontal direction, without averaging. Fifteen 0.5-mm thick slices with an interslice distance of 0.6 mm were imaged with a field of view (FOV) of 2 cm×2 cm and a matrix size of 384×384, resulting in a nominal voxel size of 52 µm×52 µm. The acquisition time was 3 min and 21 s for each direction.
2. A 3D velocity compensated GRE sequence with TE/TR = 12/250 ms, α = 15°, and no averaging was applied. A horizontal slab was acquired covering a FOV of 1.5 cm×1.2 cm×2.2 cm with matrix dimensions of 248×199×36, resulting in an isotropic resolution of 60 µm×60 µm×60 µm. The acquisition time was 29 min 51 s. Based on these GRE data magnitude images, SWI and QSM have been computed and analyzed.

### Sequence parameters for the second set of measurements performed at 4.7T

1. Anatomical reference data were acquired at 4.7 T by applying a SE sequence (RARE) with TE/TR = 33/2500 ms, RARE factor  = 8 in axial, and sagittal direction, without averaging, for planning of subsequent image acquisitions. Nine 1-mm thick slices with an interslice distance of 1.3 mm were imaged with a FOV of 2 cm×2 cm and a matrix size of 200×200, resulting in a nominal voxel size of 100 µm×100 µm. The acquisition time was 1 min and 2 s for each direction.
2. For DWI, a four-shot SE echo planar imaging (SE-EPI) sequence with TE/TR = 29.4/3000 ms was used, starting with 4 dummy scans. Ten 1-mm thick slices with an interslice distance of 1.5 mm were acquired with a FOV of 3.29 cm×2 cm and a matrix size of 128×128, resulting in a nominal voxel size of 257 µm×156 µm. Diffusion-encoding was applied in x-, y- and z-direction (gradient pulse duration  = 7 ms, gradient pulse separation  = 14 ms) with b-values of 100, 200, 400, 600, 800, and 1000 s/mm^2^, respectively. The total acquisition time was 3 min 48 s.For T_1_ mapping, six sets of inversion recovery images were collected using multiple SE sequences with slice selective RF pulses. Inversion times were 90.3, 150, 500, 1000, 3000, and 5500 ms. Imaging parameters were: TE = 8.2 ms, 3 averages and a RARE factor  = 2. The acquisition time was 15 min 21 s.
3. For DCE-MRI a fast low angle shot (FLASH) sequence with TE/TR: 4.6/12 ms, α = 15° and 3 averages was repeated 300 times. Gd-DOTA (Dotarem, Guerbet, Paris, France) was injected intravenously as a 50 µl bolus (2000 µl/min) after the 50^th^ repetition. For both T_1_-mapping and DCE-MRI a single axial slice of 2 mm with a FOV of 2 cm×2 cm and a matrix size of 80×80 was acquired, resulting in an in-plane resolution of 250 µm×250 µm. The acquisition time was 14 min 24 s.

### Immunohistochemistry

Tissue preparation, immunohistochemistry protocol, and microscopy were performed as described previously [22]. As a primary antibody a mouse anti-human Aβ1–17 monoclonal antibody (clone 6E10, SIG-39320, 1∶2000, Covance, Princeton, NJ, USA) and as a secondary antibody a goat-anti-mouse antibody (Jackson ImmunoResearch Laboratories Inc., Suffolk, GB,1∶500) were used.

### Selection of regions-of-interest

**Figure 1:**
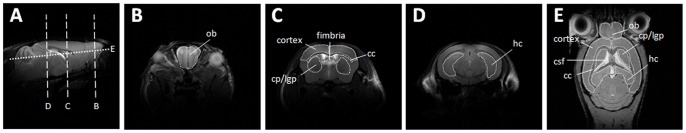
Examples on region-of-interest (ROI) delineating different anatomical regions. T_2_-weighted spin echo images of the mouse brain acquired at 9.4T in sagittal (A), axial (B–D) and horizontal (E) orientation. ROIs were identified for the olfactory bulb (*ob*), cerebral cortex (*cortex*), caudate putamen and lateral globus pallidus (*cp/lgp*), hippocampus (*hc*), corpus callosum (*cc*), fimbria hippocampi (*fimbria*) and ventricles (*csf*).

Regions-of-interest (2D; ROIs) were drawn on the parametric maps by a person blinded to the genotype. In quantitative susceptibility maps volumes-of-interest (3D; VOIs) where drawn, excluding CMBs. Where applicable, olfactory bulb (*ob*), cerebral cortex (*cortex*), caudate putamen and lateral globus pallidus (*cp/lgp*), hippocampus (*hc*), corpus callosum (*cc*), fimbria hippocampi (*fimbria*) and ventricles (*csf*) were identified (Fig. 1) as described in [32]. Mean values were calculated for 2D and 3D region. For the *cortex, cp/lgp, hc* and *fimbria*, ROIs were also drawn in each hemisphere separately and values were averaged. ROIs were in general selected to cover regions that are affected early and strongly by Aβ deposition e.g. *cortex* and regions that were affected later and to a lesser extent e.g. *cp/lgp*.

### Data processing

Apparent diffusion coefficient (ADC) maps were computed from diffusion-weighted images [33] using Paravision (Bruker BioSpin MRI). The index of diffusion anisotropy (IDA) was calculated [34] as:
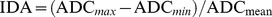
(1)where ADC_max_ corresponds to the maximum, ADC_min_ to the minimum and ADC_mean_ to the mean ADC value measured in each selected ROI.

T_1_-maps were computed pixel-wise through a non-linear least-squares fit [35]. DCE-MRI data analysis was performed as described [36] with custom software written in MATLAB (Mathworks, Natick, MA) and yielded the relative transfer constant of Gd-DOTA from the plasma into extracelluar space, K^trans^, and the relative volume of the extravascular extracellular space, v_e_.

Quantitative susceptibility maps and SW images were computed from the GRE data. SW images were generated as described previously [30], [31]. For QSM, phase images were first unwrapped using a 3D best-path algorithm [37] and background phase contributions were eliminated with projection onto dipole fields [38]. Brain tissue masks were generated, and applied for background phase correction and removal of extracranial structures. Quantitative susceptibility maps were reconstructed from the background-corrected phase images through Homogeneity Enabled Incremental Dipole Inversion [29]. Magnetic susceptibility Δχ_I_ of specific brain region I were calculated according to:
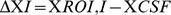



Since the calculated susceptibility values represent relative rather than absolute values [39], susceptibility values were specified as difference with respect to the magnetic susceptibility measured in the ventricles. We have excluded Aβ-related effects on the magnetic susceptibility of the reference region by performing an analysis of covariance between the values of magnetic susceptibilities measured in the ventricles of arcAβ and wt mice. We found no significant differences between the slope and the intercept of the two regression lines (F = 0.28, p = 0.59 and F = 0, p = 1, respectively).

### Assessment of CMB load

CMBs were identified as round or ovoid lesions on horizontal GRE magnitude and SW images (black lesions) as well as quantitative susceptibility maps. Additional slices above and below the slice of interest were viewed, to ensure that the suspected CMB was confined and not a vessel cross-section. Each identified CMB was encircled by a VOI. A template was generated from GRE magnitude images by non-linear incremental registration using advanced normalization tools [40]. CMBs were then superposed onto the registration template image by applying the computed affine registration matrices and deformation fields to each VOI of the CMB with nearest neighbour interpolation. VOIs for each sample volume were manually counted to estimate CMB load. CMB load among groups was compared using Fisher's exact test.

### LME modelling of MRI data

Analysis was performed with the statistical computing environment R [41]. The LME model was fitted for each MRI parameter (Y) according to:
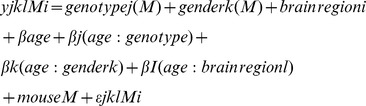
(2)

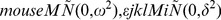
(3)where 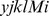 represents the measurement i of MRI parameter Y for mouse M with genotype j and gender k in brain region i. The nested design (i.e. each mouse belongs to one genotype and has one gender) is denoted by the functional index of M. The terms 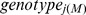, 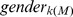 and 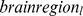 are the fixed effects of genotype, gender and brain region which are assumed to be identical for all mice. The coefficient β describes the slope in age. Since the slope of time might depend on the brain region, gender and genotype, we further included interactions (denoted with colons, e.g. age*genotype). 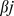 represents the interaction of the slope in age by the 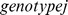,, 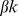 by 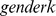, and 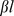 by 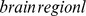. The random effect 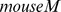 represents the deviation for each mouse M from the population mean. It is modelled as normally distributed random variable and incorporates the correlation of the repeated measurements in its variance. 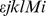 is the usual error term for observation *i*. Model assumptions were checked by residual analysis (QQ-plot, Tukey-Anscombe-plot). To identify differences in brain regions *post hoc* tests were performed. In case of more than two brain regions, p-values were corrected by the Bonferroni method. A value of p<0.05 was considered as statistically significant.

## Results

### Characteristics of longitudinal data

The number of animals decreased over the course of the study (Table 1). Animals whose physiological conditions were compromised, e.g. due to spontaneous tumor development in aged mice, had to be excluded. Moreover, each scan session (including animal preparation and measurement) lasted about 1 hour, during which animals were anesthetized. These anesthesia episodes also caused drop-out of animals, in particular in aged mice. In addition, missing data occurred as animals were not scanned due to unforeseen problems in animal preparation or if data had to be excluded due to insufficient quality.

A second characteristic of longitudinal data is the non-uniform timing of assessments. Animals differ in age by a few days when entering the study (Table 1), and hence are not assessed at exactly the same time point. In addition, given the large number of animals assessed at each scan session, measurements with an individual set-up can take up more than one week, leading to variations in the time intervals between scans for individual animals.

A third characteristic of the data is the between-subject variability of values of MRI parameters (Table S1).

### LME modelling of structural MRI parameters

**Figure 2:**
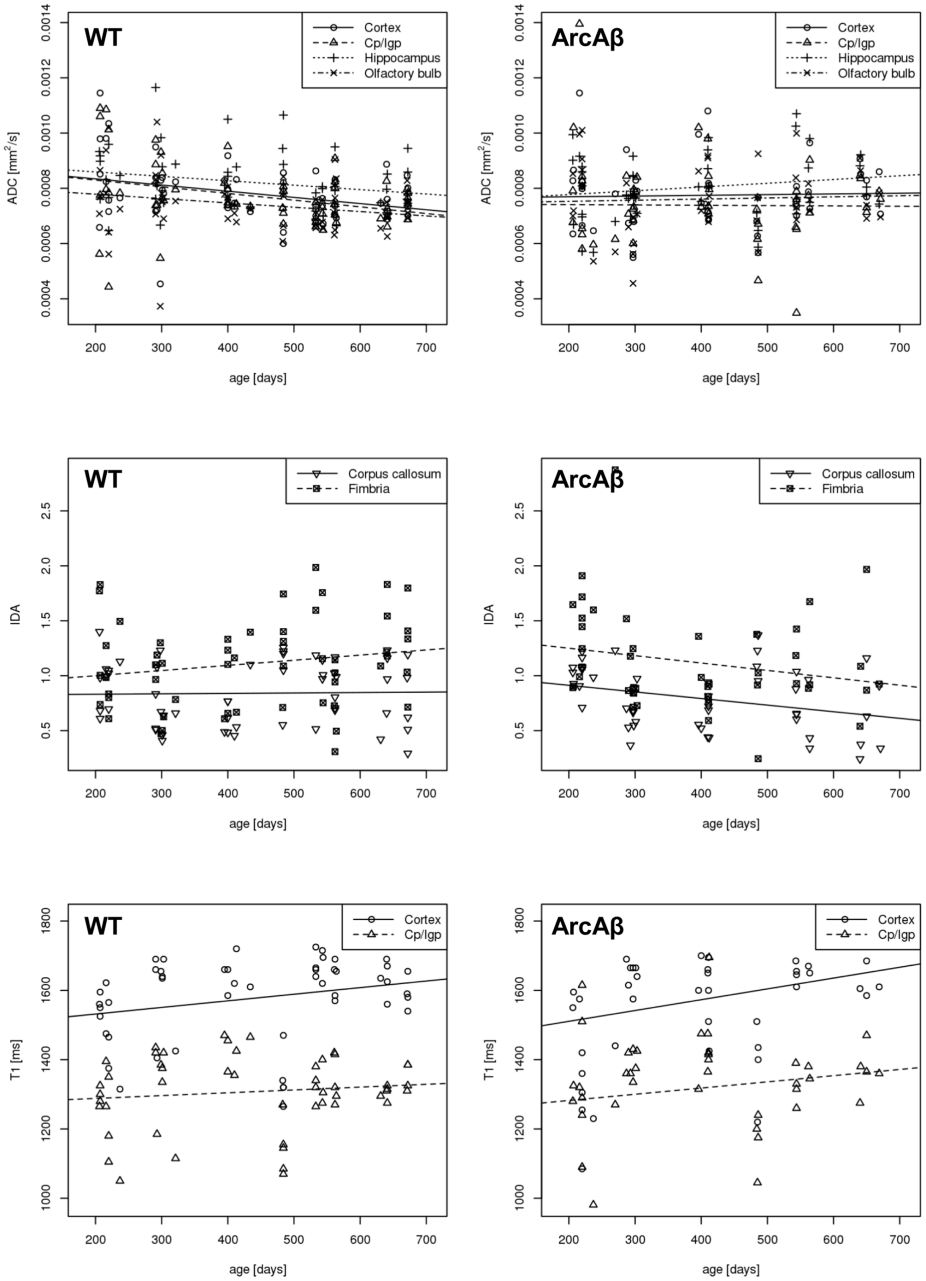
Scatter plots of apparent diffusion coefficient (ADC), index of diffusion anisotropy (IDA) and T_1_ relaxation times as a function of age for wild type (WT) and transgenic arcAβ (ArcAβ) mice. *Cp/lgp* denotes caudate putamen and lateral globus pallidus.

The results of the LME model analysis demonstrated that ADC values of grey matter regions were significantly associated with brain region, gender and age as well as interaction of genotype and age (Table 2). *Post hoc* analysis revealed that the ADC values of *hc* were significantly different from the ADC values of *cp/lgp* and *ob*, but not from those observed in *cortex* (Table 3). The evolution of ADC with age was significantly different between arcAβ and wt mice. (Fig. 2). While ADC decreased in different regions in wt mice with increasing age, it stayed constant or even tended to increase in arcAβ mice (slope  =  −8×10^−5^ mm^2^/s per year in wt vs 1.89×10^−5^ mm^2^/s per year in arcAβ mice). IDA values of two white matter regions were significantly associated with brain region, and the interaction of genotype and age (Table 2). Values were higher for *fimbria* compared to *cc* (Fig. 2, Table 3). Moreover, for both regions IDA values developed differently in wt and arcAβ mice as a function of age. They slightly increased in wt mice, but decreased in arcAβ mice, indicating a loss of diffusion anisotropy in the latter (slope  = 0.0741 for wt vs slope  =  −0.3134 for arcAβ mice; Fig. 2).

**Table 2:** P-values of linear mixed effects model analyses of the MRI parameters.

	ADC	IDA	T_1_	magnetic susceptibility	K^trans^	v_e_
brain region	**0.0002**	**<0.0001**	**<0.0001**	**<0.0001**	**0.0473**	0.1906
genotype	0.3913	0.9186	0.7974	**0.0367**	0.1822	0.3336
age	**0.0157**	0.3781	**<0.0001**	**<0.0001**	**0.0112**	**0.0110**
gender	**0.0010**	0.8306	0.5247	0.7041	0.1688	0.6203
brain region:age	0.7354	0.5539	0.1608	0.1305	0.7535	0.6826
genotype:age	**0.0005**	**0.0023**	0.5137	**0.0159**	0.1953	0.7049
gender:age	0.0619	0.5379	0.0570	**0.0002**	0.3443	0.6463

ADC values were determined for *ob*, *cortex*, *cp/lgp*, and *hc*. IDA values were estimated for the *cc* and *fimbria*. T_1_, K^trans^ and v_e_ were estimated for the *cortex* and *cp/lgp*. Magnetic susceptibility values were estimated for *ob, cortex, cp/lgp, cc,* and *hc*. Statistical significance is highlighted (bold).

**Table 3:** *Post hoc* analysis assessing different values of individual MRI parameters between brain regions.

MRI parameter		p-value
**ADC**	hc vs. cortex	0.1049
	hc vs. cp/lgp	0.0011
	hc vs. ob	0.0003
	cortex vs. cp/lgp	0.9828
	cortex vs. ob	0.4837
	cp/lgp vs. ob	1.0000
**IDA**	fimbria vs. cc	<0.0001
**T_1_**	cortex vs. cp/lgp	<0.0001
**magnetic susceptibility**	cc vs. cortex	<0.0001
	cc vs. cp/lgp	<0.0001
	cc vs. hc	<0.0001
	cc vs. ob	<0.0001
	cortex vs. cp/lgp	1
	cortex vs. hc	0.197
	cortex vs. ob	1
	cp/lgp vs. hc	0.022
	cp/lgp vs. ob	1
	hc vs. ob	0.104
**K^trans^**	cortex vs. cp/lgp	0.0473
**v_e_**	cortex vs. cp/lgp	0.1906

ADC values were determined for *ob*, *cortex*, *cp/lgp*, and *hc*. IDA values were estimated for the *cc* and *fimbria*. T_1_, K^trans^ and v_e_ were estimated for the *cortex* and *cp/lgp*. Magnetic susceptibility values were estimated for *ob, cortex, cp/lgp, cc,* and *hc*. Statistical significance is highlighted (bold).

T_1_ relaxation times were significantly different between *cortex* and *cp/lgp* and revealed an association with age, but neither with genotype nor the interaction of genotype with age (Table 2 and 3). Trajectories of T_1_ values increased similarly in both groups over time with (Fig. 2).

**Figure 3:**
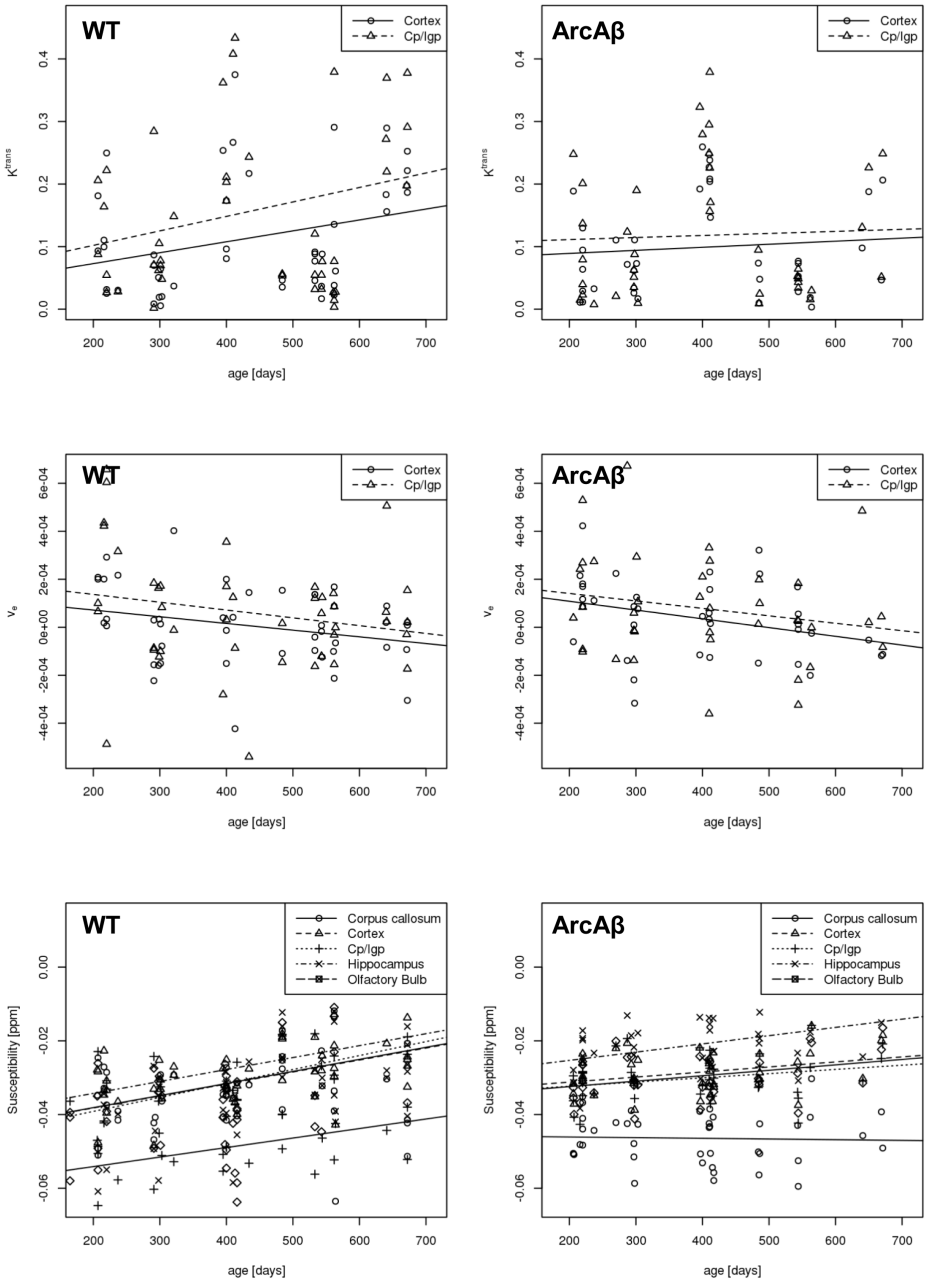
Scatter plots of K^trans^, v_e_ and magnetic susceptibility as a function of age for of wild type (WT) and arcAβ (ArcAβ) mice. *Cp/lgp* denotes caudate putamen and lateral globus pallidus.

Magnetic susceptibility was found to be significantly associated with brain region, age and genotype as well as the interaction of genotype with age and gender (Table 2). *Post hoc* analysis revealed that the values in *cc* and *hc* were significantly different from each other and from those of all other brain regions (Table 3). Susceptibility differences were lower for the *cc* and higher for the *hc* compared to all other brain regions investigated. Magnetic susceptibilities increased in different regions of the brain in both groups with age, but the slope was smaller in arcAβ mice compared to wt mice (slope  = 1.04×10^−2^ for male and slope  = 2.92×10^−3^ for female wt mice vs slope  = 6.52×10^−3^ for male and slope  =  −9.21×10^−4^ for female arcAβ mice; Fig. 3).

### LME modelling of vascular MRI parameters

K^trans^ and v_e_ values showed an association with age, but neither with genotype nor interaction of genotype with age (Table 2). K^trans^ was also associated with brain regions were significant different values were observed between *cortex* and *cp/lgp* (Table 3). K^trans^ increased with age, whereas v_e_ decreased in all brain areas evaluated (Fig. 3).

**Figure 4:**
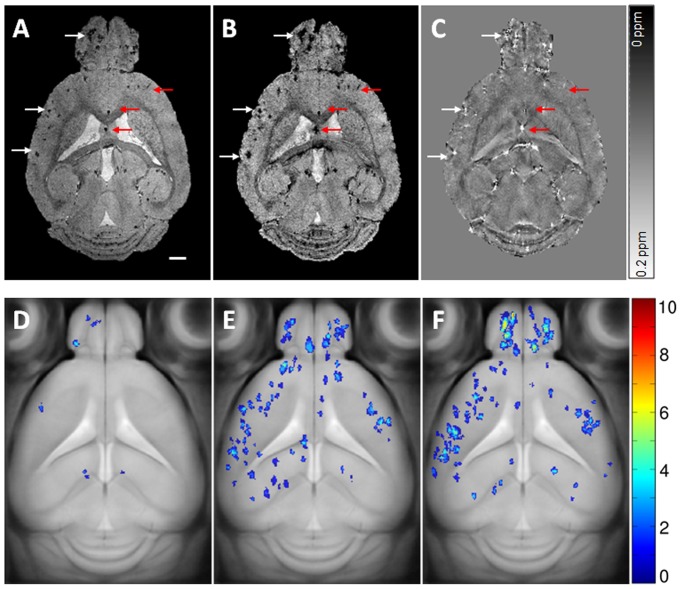
Horizontal GRE magnitude images, susceptibility weighted images and quantitative susceptibility maps of a 21 month old arcAβ mouse. Suspected microbleeds are indicated by white arrows, while structures corresponding to vessel cross-sections are indicated by red arrows. The scale bar indicates 1 mm. Topography of cerebral microbleeds (CMBs) of a an arcAβ mouse with high CMB load at 13 (A), 18 (B) and 21 (C) months of age. Average projections of the registration template over an acquired slab of 2.1 mm thickness. The brain regions affected are predominantly the cortex and olfactory bulb and to a lesser extent the hippocampus. The CMB load increases with increasing age. The frequency of overlapping CMBs in 3D assessed at a single time point is indicated by the colour bar.

SWI and QSM have increased sensitivity for detecting CMBs compared to conventional GRE magnitude images [22]. CMBs were identified as round or ovoid lesions on horizontal GRE magnitude and SW images (black lesions) as well as quantitative susceptibility maps (Fig. 4A–C, white arrows). The load of paramagnetic inclusions, attributed to CMBs, was markedly higher in arcAβ mice compared to wt mice (Table 4). Probable CMBs were first observed in arcAβ mice at 9 months of age, whereas they became first evident in wt mice at 13 months. CMBs were detected in 71% of arcAβ mice at 18-month of age, but only in 20% of wt mice (at 21 month of age). The total number of CMBs detected in arcAβ mice was significantly higher in arcAβ mice than in wt mice (p<0.001). CMBs were predominantly observed in the *cortex* and *ob*, but were also seen in the *hc* (Fig. 4).

**Table 4:** Occurrences of cerebral microbleeds (CMBs).

		wt			arcAβ	
**age [months]**	**animals**	**animals with CMBs**	**CMBs/mouse (95% CI)**	**animals**	**animals with CMBs**	**CMBs/mouse (95% CI)**
**scan 1**	9	0	0	11	0	0
**scan 2**	8	0	0	6	2	1 (1)
**scan 3**	17	1	2	16	10	9 (4–14)
**scan 4**	10	2	2 (0–8)	7	5	26 (0–65)
**scan 5**	5	1	2	3	1	96

CMBs were identified from GRE magnitude images, susceptibility weighted images and quantitative susceptibility maps. The number of animals, the incidence (animals with CMBs) and mean number of CMBs, 95% confidence interval (CI) per affected mouse detected in wild type (wt) and transgenic arcAβ mice at different ages.

### Age-dependent deposits of Aβ plaques in arcAβ mice

**Figure 5:**
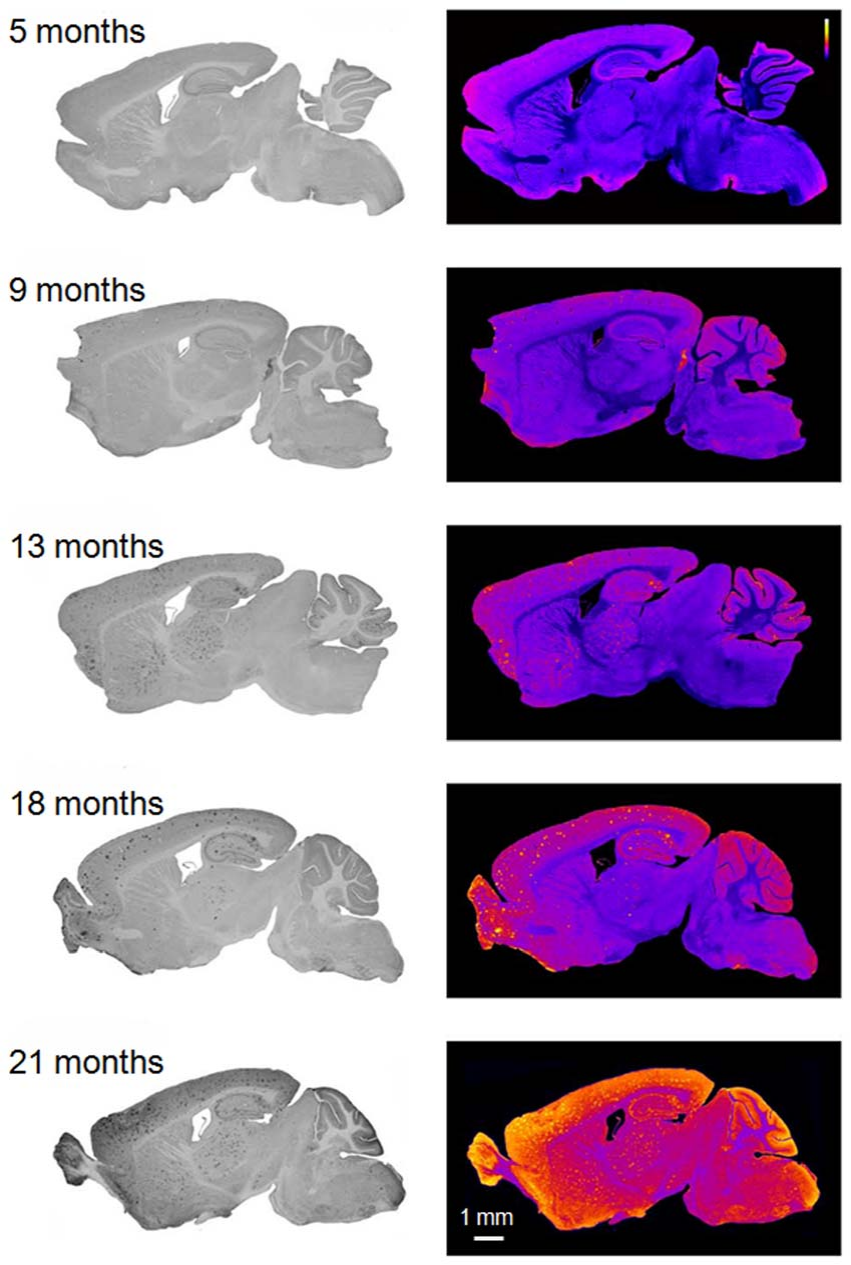
Age-dependent accumulation of Aβ plaques in arcAβ mice. Anti-Aβ immunohistochemical staining of sagittal brain sections at 5, 9, 13, 18 and 21 months of age. Right panels show normalized colour-coded images depicting the strongest signals in yellow and background in dark blue. Animals at 6 months of age were devoid of plaques, initial plaque formation in neocortical areas became apparent at 7 months of age. Plaques rapidly increase in number between 13 and 21 months of age, affecting hippocampus as well as striatal and thalamic areas. Late stages also included plaque deposition in the cerebellum and brainstem areas. The scale bar indicates 1 mm.

Immunohistochemical evaluation revealed an age-dependent increase in Aβ plaque deposition in arcAβ mice (Fig. 5). Animals at 6 months of age were devoid of plaques, initial plaque formation in neocortical areas became apparent at 7 months of age. Plaque deposition increased until an age of 21 months and spread to hippocampal, striatal, thalamic as well as to cerebellar and brainstem regions.

## Discussion

### Characteristics of longitudinal MRI data

One of the main challenges of longitudinal studies is missing data. Not all mice in our study have data on all 5 assessments. This is either due to design of the study (one batch entered at scan session 3) or due to premature demise of animals which becomes in particular weighty towards the end of the study (Table 1) as aged animals became increasingly fragile. In addition, a few animals missed a particular measurement but were assessed during the next scan session. Also data had to be excluded from the analysis due to insufficient quality. A major drawback of missing data is that conventional methods for statistical analysis e.g. repeated measure ANOVA are not applicable i.e. it could be applied only to few cases were 5 complete measurements are available for an animal. Imputation methods for missing data have been developed but they have limitations [42]. We have opted to use LME modeling as a statistical framework because it draws trajectories across a population rather than individuals and can account for missing data [24], [25]. Thus the number of animals per group analyzed were n = 15 (wt) and n = 17 (arcAβ) for ADC and IDA, n = 15 (wt) and n = 17 (arcAβ) for T_1_, n = 19 (wt) and n = 21 (arcAβ) for magnetic susceptibility, and n = 15 (wt) and n = 15 (arcAβ) for K^trans^ and v_e_.

A second important feature of longitudinal data sets is that they are unbalanced with regard to the measurement time. Animals cannot be assessed at once within a single scan day. In fact, the assessment of the first and last animal in a single scan session can vary by as much as by two weeks. These variances in measurement time also preclude the use of repeated measure ANOVA. In contrast, LME model is suitable to handle such unbalanced data sets [24], [25].

In this study we used mice of the same strain with a controlled genetic background. Hence, phenotypes were expected to be homogenous. However, we observed a high degree of between-subject variability of investigated MRI parameters (Table S1). Factors such as age, gender and environment have been reported to affect the phenotype [43]. While some of these factors can be controlled for e.g. breeding and housing conditions of mice, other factors (keeping single mouse in cage vs. housing in groups, individual degrees of physical exercise, fighting among animals, comorbidities etc.) cannot be controlled and might thus account for the observed variability.

### LME modelling reveals effects unrelated to disease pathology

In the LME model we have included the parameters brain region, gender and age as fixed variables to account for changes unrelated to disease pathology. The analysis revealed that almost all parameters, except v_e_, depended on the brain regions. This is not surprising, given that the cellular morphology, myo- and cytoarchitecture, water content and diffusivity, concentration of minerals, vascular density, and metabolic activity of the brain are known to differ between anatomical regions. In addition, the analysis revealed an effect of gender on ADC and an interaction of age and gender on magnetic susceptibility. While sexual dimorphism of the mouse brain has been reported for volumes of certain brain structures [44] it was not known to what extent it might affect the MRI parameters investigated in this study. To date, most MRI studies using mice have not looked for differences between gender and often do not even report whether male, female or mice of both genders were used.

Our analysis revealed that aging had significant effects on all parameters investigated, except IDA. Aging leads to changes in tissue structure and composition as well as vascular function [45]–[48]. These changes clearly affect the MRI parameter under study and in fact the mechanism of these changes might resemble those seen during pathological states. Hence, imaging approaches need to be capable to identify pathological changes from age-related processes.

### LME model demonstrates an effect of pathology on water diffusivity and magnetic susceptibility over time

Subsequently, we used LME modelling of data to reveal which parameters were significantly different in arcAβ mice compared to wt mice. An effect of genotype was observed only on magnetic susceptibility. But accepting the interaction of age and genotype, the structural parameter ADC, IDA and magnetic susceptibility were significantly different in arcAβ mice compared to wt mice. Previous studies have reported changes in water diffusivity in the brain of transgenic mice. Decreased ADC values were observed in APP23 mice compared to age-matched wt controls [12], [13], while a study in TgCRND8 mice found no significant changes of ADC in the *hc* and *cortex* [14]. Mueggler et al. found ADC values in cortex and striatum of 6 month old APP23 mice ADC to be between 5.47±0.25 and 6.91±0.22 and 5.90±0.19×10^6^ cm^2^/s, respectively [12]. In the cortex and striatum of 25 months old APP23 mice ADC values were found to be between 5.09±0.33 and 6.86±0.34 and 5.79±0.43×10^6^ cm^2^/s for, respectively. Sykova et al. reported ADC values to be between 5.96±0.07 and 5.97±0.15×10^6^ cm^2^/s in the brains of 6–8 month and between 5.74±0.09 and 5.89±0.03×10^6^ cm^2^/s in the brains of 17–25 months old male and female APP23 mice, respectively [13]. White matter regions were not assessed in these studies. These findings are clearly in contrast to our study where an increase in grey matter ADC values and a decrease in IDA values were observed in arcAβ mice (Fig. 2). Moreover, ADC values were with 8.22±1.36 and 8.23±2.39×10^6^ cm^2^/s in the *cortex* and *cp/lpg* of 5 month old arcAβ mice generally higher than those reported for APP23 of similar age (Table S1). Similarly, ADC values were 8.21±0.65 and 7.83±0.50 in the *cortex* and *cp/lpg* of 21 month old arcAβ mice and thus higher as the reported values in APP23 mice. The differences in values might be explained in parts by differences in methodology since we have also observed higher ADC in the brains of wt mice. However, water diffusivity might differ among different transgenic mouse lines and at different ages. The observed lack of differences in ADC values between 12–16 month old TgCRND8 mice [14] and 6–8 month old APP23 mice [12], [13] and age matched controls might be a consequence of still relatively mild amyloid pathology. On the other hand, the observation of reduced ADC values in 25 month old APP23 animals might represent an effect of end-stage pathology (an age which we have not assessed in arcAβ mice). All of those studies were performed in cross-sectional design comparing two age groups (though some of them spanning several months of age) and thus the reported differences represent only a snapshot or averages of values. Information about how a parameter has evolved in-between or after an early assessment is missing. Using longitudinal data we observed unchanged or slightly increasing ADC values in arcAβ mice between 5 and 21 month of age. In future studies we would use diffusion tensor imaging which information about the fractional anisotropy in addition to the diffusion coefficient.

Studies have also examined the effect of amyloid-related pathology on the tissue relaxation times of the brain. While Helpern et al. have found no differences in T_1_ values between different brain regions of 16–23 month old APP/PS1 and PS1 and non-transgenic controls [15], El Tannir El Tayara et al. reported a significant differences between the brain of APP/PS1 and PS1 mice when measured over time from 27 to 86 weeks of age [16]. T_1_ values were approximately 1275 ms in the cortex and 1285 ms in the striatum of 28 week old APP/PS1 mice and decreased to approximately 1210 ms and 1190 ms in the cortex and striatum in 80 weeks old APP/PS1 mice, respectively. T_1_ values in the study of Helpern et al. were not given. In 5 month old mice arcAβ mice we measured T_1_ values of 1464±478 and 1226±400 ms for the *cortex* and *cp/lgp*, respectively (Table S1), thus being similar to reported values. T_1_ increased to 1603±616 and 1322±506 ms for the *cortex* and *cp/lpg* of 21 month old arcAβ mice, respectively. However, T_1_ values increased also in wt mice and we observed no differences between genotypes. The difference in T_1_ among different mouse strains might be explained by the fact that plaques in APP/PS1 mouse brains contain iron [15] leading to decreases in T_1_ of the tissue with accumulation of plaques. In contrast, cortical and subcortical plaques in the arcAβ mice contain no iron [22]. Thus, plaque deposition does not seem to affect T_1_.

### Effects of amyloid-related pathology on vascular function

As vascular pathology has been implicated to partake in the pathogenesis of the disease, we have additionally used MRI techniques which are sensitive to changes in vascular function. The arcAβ mouse strain used, which expresses both the Swedish and Arctic APP mutations [26], shows both amyloid-related changes in brain parenchyma and at the cerebral vasculature. In this strain Aβ deposition starts at around 7 months of age and is not only confined to the brain parenchyma but occurs also at cerebral blood vessels [6], [26]. Aβ deposition starts in the cerebral cortex and then spreads to the dorsal and ventral areas of the brain with age (Fig. 3; [26]). Aβ deposition is accompanied by astrogliosis in particular surrounding diffuse Aβ plaques in the neutropil and around CAA-affected vessels [6]. Vascular pathology related to amyloid is pronounced and includes changes in vessel morphology, impairment of vascular reactivity, vascular fibrinogen deposition and vessel stenosis, neurovascular uncoupling, loss of vascular smooth muscle cells with BBB leakage and the occurrence of CMBs [6], [20]–[22].

We have probed BBB integrity and occurrence of CMBs in arcAβ mice with MRI. Using DCE-MRI we did not find signs of evident BBB leakage which is surprising as a compromised BBB integrity of Aβ-affected vessels has been described for this mouse strain, detecting the extravasation of a vascular fluorescent marker [6]. One explanation might be that BBB dysfunction in AD is more subtle compared to diseases such as brain tumors, multiple sclerosis and stroke, for which the impairment is relatively large and focal [49]. Hence, in contrast to histological techniques DCE-MRI may not be sufficiently sensitive to detect BBB impairment in mouse models of AD in-vivo.

We have shown previously that GRE imaging can be applied to non-invasively detect CMBs in the mouse brain [22]. CMBs were found to occur frequently with age in arcAβ mice. Studies have also demonstrated that CMBs also occur in other mouse strains like APP23 and Tg2576 mice [50], [51]. CMB incidence and load increased between 9 and 18 month of age (Table 4), but drop-outs of animals during the study render it difficult to judge how this progressed beyond 18 month of age. We found the CMBs mainly affected *ob, cortex* and *hc*. However, due to time constraints associated with high spatial resolution measurements (60 µm isotropic voxels) the assessment of CMBs was limited to a 2.2 mm slab covering essentially cortical structures only. Hence, we cannot make a statement regarding the incidence of CMBs in more basal brain areas. Given that the incidence of CMBs in arcAβ mice was between 33 and 71% during 9 to 18 month while they were 0 to 20% in wt mice during the same age span, it appears that detection of CMB load is a sensitive indicator of vascular pathology.

### The limitations of using MRI for detecting amyloid-related pathology in transgenic mouse models of AD

We have found a significant interaction of age and genotype for QSM and DWI, both techniques may be associated with pathological changes in tissue structure and composition. It is known that many processes such as glia activation, de- and remyelination and impairment of membrane integrity can affect water diffusivity and magnetic susceptibility [33], [52], but it was not the purpose of this study to evaluate the contrast mechanism underlying the changes in these parameters in the arcAβ mouse brain. Such studies would have required sacrificing animals after MRI measurement in order to perform biochemical analysis and histology of the brain, which was precluded in the longitudinal design chosen. Moreover, since water diffusion and tissue magnetic susceptibility are affected by a variety of processes/sources which change the biophysical properties of the tissue, these methods will never be entirely specific for a pathological process but rather reflect the sum of microstructural changes occurring in the tissue. Only in the best case the values are governed by a single dominating mechanism.

## Conclusions

MRI studies of transgenic mouse models are affected by variability of disease phenotype, making the detection of subtle and slowly developing effects of pathology on MRI parameter challenging. Under these conditions, a longitudinal study design where data is pooled across the study population enables the identification of changes in MRI parameters that can be attributed to the pathology. LME modelling is a suitable framework for analyzing longitudinal data sets as it accounts for confounders unrelated to AD pathology which cannot be easily controlled for in such studies.

## Supporting Information

Table S1 **Mean values±standard deviations have been determined for individual MRI parameter for each genotype for each set of measurements.**
(DOCX)
