# Supplementary material for: Longitudinal Assessment of Amyloid Pathology in Transgenic ArcAβ Mice Using Multi-Parametric Magnetic Resonance Imaging
Source: PLoS One. 2013 Jun 19;8(6):e66097. doi: 10.1371/journal.pone.0066097 (PMC3686820; doi:10.1371/journal.pone.0066097)
Supplement: Table S1 — Mean values±standard deviations have been determined for individual MRI parameter for each genotype for each set of measurements. (DOCX) [file pone.0066097.s001.docx]

**Table S1 Values for MRI parameters**

| **wt** | **scan1** | **scan2** | **scan3** | **scan4** | **scan5** |
| --- | --- | --- | --- | --- | --- |
| **ADC*** |  |  |  |  |  |
| cortex | 8.77±1.54 | 7.78±1.19 | 7.66±0.88 | 7.51±0.59 | 7.69±0.63 |
| ob | 7.83±1.34 | 7.65±1.58 | 7.38±0.65 | 7.22±0.80 | 7.18±0.56 |
| cp/lpg | 8.41±2.23 | 7.98±1.1 | 7.67±0.69 | 7.38±0.74 | 7.44±0.56 |
| hc | 8.33±1.03 | 8.45±1.28 | 8.59±1.07 | 7.67±0.73 | 7.89±0.71 |
| **IDA** |  |  |  |  |  |
| fimbria | 1.09±0.41 | 0.98±0.32 | 1.11±0.35 | 1.08±0.49 | 1.29±0.34 |
| cc | 1.36±0.54 | 0.98±0.53 | 1.11±0.61 | 1.32±0.44 | 1.19±0.66 |
| **T_1_^#^** |  |  |  |  |  |
| cortex | 1505±97 | 1544±157 | 1506±167 | 1631±104 | 1587±105 |
| cp/lpg | 1255±102 | 1281±154 | 1296±157 | 1315±88 | 1301±84 |
| **ΔΧ_I_** |  |  |  |  |  |
| cp/lpg | -0.037±0.010 | -0.033±0.009 | -0.031±0.008 | -0.022±0.014 | -0.025±0.007 |
| cc | -0.050±0.010 | -0.051±0.007 | -0.051±0.007 | -0.041±0.016 | -0.044±0.006 |
| cortex | -0.035±0.010 | -0.034±0.008 | -0.033±0.007 | -0.022±0.015 | -0.027±0.008 |
| hc | -0.031±0.011 | -0.028±0.009 | -0.028±0.007 | -0.020±0.010 | -0.020±0.008 |
| ob | -0.034±0.010 | -0.036±0.007 | -0.030±0.012 | -0.024±0.016 | -0.030±0.006 |
|  |  |  |  |  |  |
|  |  |  |  |  |  |
| **arcAβ** | **scan1** | **scan2** | **scan3** | **scan4** | **scan5** |
| **ADC*** |  |  |  |  |  |
| cortex | 8.22±1.36 | 7.28±1.15 | 8.18±1.37 | 7.45±0.96 | 8.21±0.65 |
| ob | 8.44±1.05 | 6.6±1.15 | 7.86±0.89 | 7.69±1.10 | 7.79±0.82 |
| cp/lpg | 8.23±2.39 | 7.28±0.90 | 8.15±1.14 | 6.83±1.46 | 7.83±0.50 |
| hc | 7.98±1.27 | 7.66±0.85 | 8.39±0.77 | 8.05±1.51 | 8.18±0.68 |
| **IDA** |  |  |  |  |  |
| fimbria | 1.31±0.35 | 1.17±0.58 | 0.91±0.22 | 1.06±0.37 | 1.07±0.47 |
| cc | 1.40±0.31 | 0.93±0.41 | 0.80±0.33 | 1.13±0.51 | 0.89±0.66 |
| **T_1_^#^** |  |  |  |  |  |
| cortex | 1464±478 | 1550±489 | 1603±516 | 1527±461 | 1603±616 |
| cp/lpg | 1226±400 | 1304±416 | 1375±445 | 1251±372 | 1322±506 |
| **ΔΧ_I_** |  |  |  |  |  |
| cp/lpg | -0.031±0.007 | -0.027±0.006 | -0.028±0.056 | -0.028±0.081 | -0.023±0.055 |
| cc | -0.044±0.006 | -0.045±0.006 | -0.047±0.007 | -0.046±0.009 | -0.043±0.005 |
| cortex | -0.029±0.005 | -0.028±0.007 | -0.029±0.006 | -0.026±0.0006 | -0.022±0.0005 |
| hc | -0.025±0.008 | -0.020±0.006 | -0.020±0.006 | -0.018±0.008 | -0.014±0.009 |
| ob | -0.031±0.005 | -0.029±0.005 | -0.030±0.005 | -0.028±0.006 | -0.024±0.006 |

Mean values±standard deviations have been determined for individual MRI parameter for each genotype for each set of measurements *ADC values x10^6^ cm^2^/s; ^#^T_1_ values in ms
